# Conformational Landscape of NADH and Ion Binding in Water/DMSO Mixtures via ^31^P NMR Spectroscopy

**DOI:** 10.1021/acs.jpcb.4c03162

**Published:** 2024-07-17

**Authors:** Jiaqi Lu, Florin Teleanu, Huijing Zou, Chengtong Zhang, Andrew Hollingsworth, Alexej Jerschow

**Affiliations:** † Department of Chemistry, New York University, New York, New York 10003, United States; ‡ Interdisciplinary School of Doctoral Studies, University of Bucharest, Bucharest 010041, Romania; § Biophysics and Biomedical Application Laboratory, Extreme Light Infrastructure Nuclear Physics, IFIN-HH, Măgurele 77125, Romania; ∥ Department of Physics, New York University, New York, New York 10003, United States

## Abstract

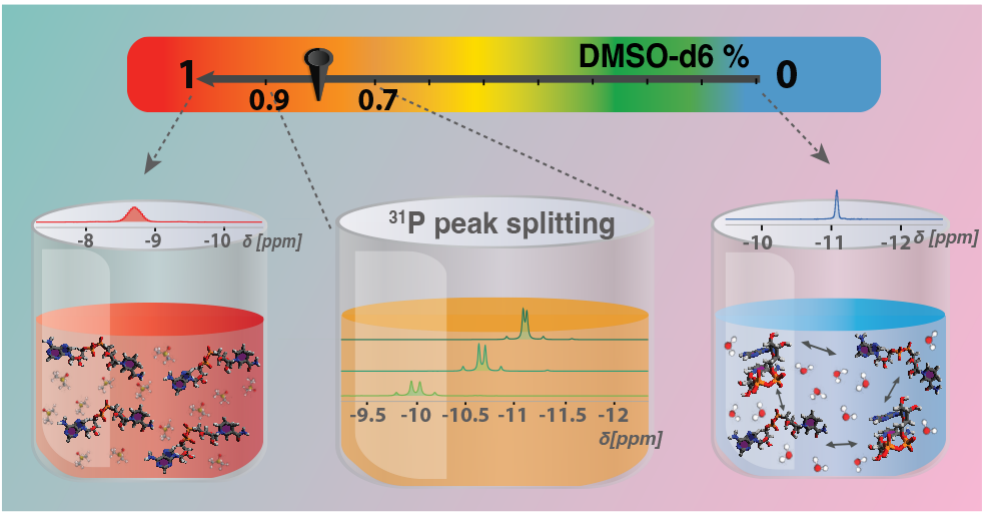

This study reports on the conformational states of nicotinamide adenine dinucleotide (NADH) in water/DMSO mixtures and examines the influence of ion binding. We observe evidence of conformational changes through a series of NMR techniques, including ^31^P NMR relaxation (*R*_1_ and *R*_2_), chemical exchange saturation transfer (CEST), and diffusion-ordered spectroscopy (DOSY) experiments. The observed variation of the conformational states is indicative of the solvent’s influence on NADH’s structural flexibility. The experimental findings, in combination with viscosity data, are shown to be in line with findings from earlier molecular dynamics studies. The reported observations emphasize the critical role of the solvent environment in determining the conformational states of NADH and similar molecules with relevance for the biophysiological context. The results found herein can help in studying the biophysical behavior of NADH and similar biomolecules and their associated metabolic pathways under various solvent conditions.

## Introduction

**Figure 1:**
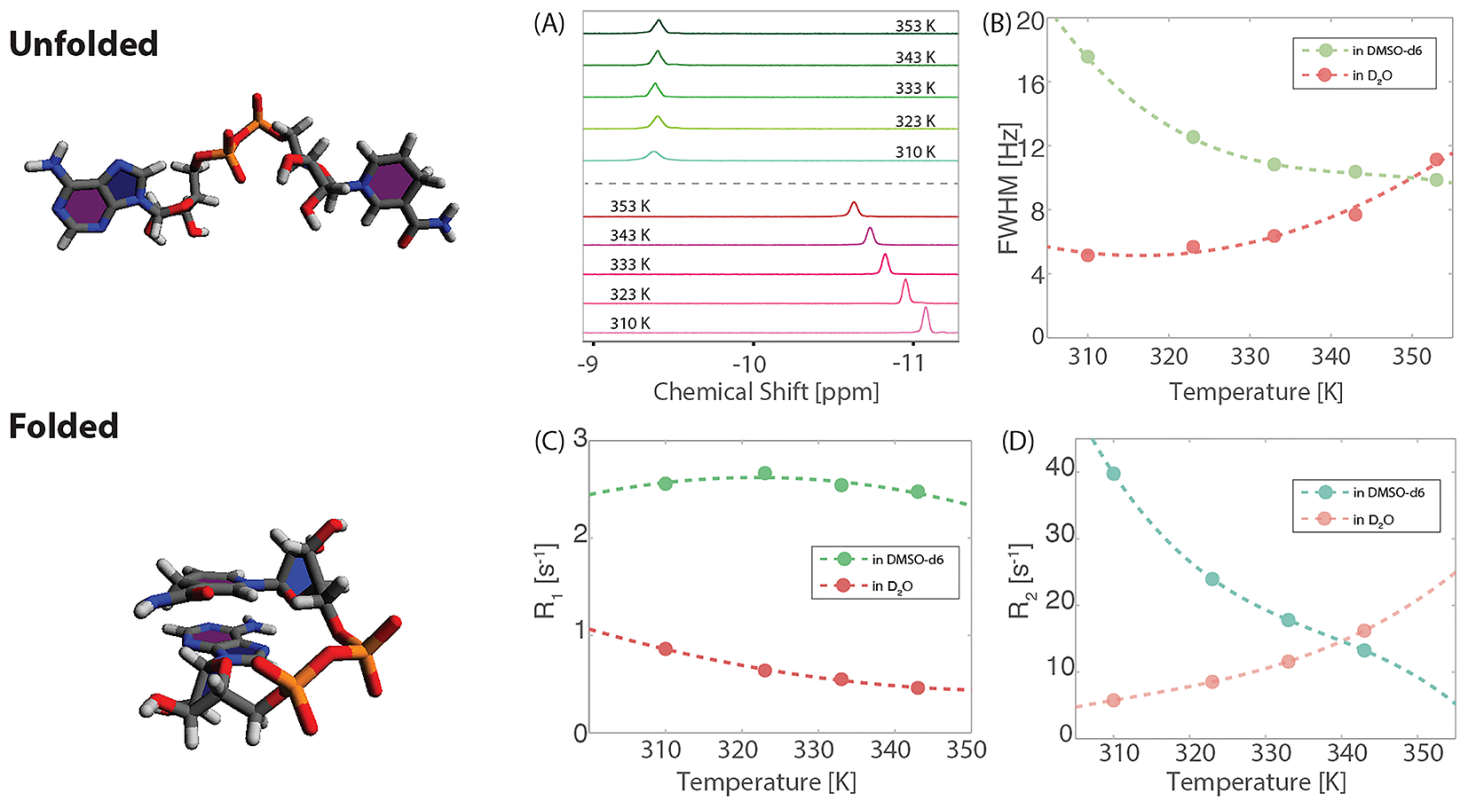
Two conformations of NADH marked as “Unfolded” and “Folded” alongside the temperature dependence of ^31^P NMR results for 30 mM NADH in pure deuterated solvents: DMSO-*d*_6_ and D_2_O. (A) Below the dashed line: 1D NMR spectra of 30 mM NADH in pure D_2_O at temperatures ranging from 310 to 353 K, demonstrating line broadening with increasing temperature. Above the dashed line: 1D NMR spectra of 30 mM NADH in pure DMSO-*d*_6_ over the same temperature range, exhibiting line narrowing as temperature increases. (B) Line widths for NADH as a function of temperature, displaying a monotonic increase in pure D_2_O and a monotonic decrease in pure DMSO-*d*_6_. (C) Spin–lattice relaxation rate (*R*_1_) as a function of temperature, showing a monotonic decrease in pure D_2_O and a local maximum in pure DMSO-*d*_6_. (D) Spin–spin relaxation rate (*R*_2_) as a function of temperature, following the same trend as the line widths in (B): a monotonic increase in D_2_O and a monotonic decrease in DMSO-*d*_6_.

Nicotinamide adenine dinucleotides (NAD) are coenzymes present in the intracellular space and form a redox equilibrium between oxidized (NAD^+^) and reduced (NADH) states. In mitochondria, they serve in metabolic processes such as the tricarboxylic acid cycle and the electron transport system where NADH assists in producing adenosine triphosphate (ATP).^1^ Both coenzymes are involved in a chemical exchange between two molecular conformations, known as “folded” (F) and “unfolded” (U) as depicted in Figure 1.^2^ Recent spectroscopic and structural evidence point toward a preference for the unfolded state when binding to the active sites of enzymes which allows for stronger host–guest interactions due to the increased molecular surface.^3−7^ As a result, perturbations of the freely available, unbounded U/F species arising from temperature,^8,9^ pH,^10,11^ solvent,^7,12^ or chemical gradients^13,14^ can impact the global enzymatic rates of energy metabolism leading to cellular malfunction. Fluorescence and nuclear magnetic resonance (NMR) spectroscopy have been used to study conformational exchange on time scales spanning several orders of magnitude by monitoring the lifetimes of nonequilibrium electronic or nuclear spin states.^15,16^ Previous studies investigated the changes in time-resolved fluorescence emission^17−19^ and/or the NMR spectral changes^20−23^ of NAD^+^/NADH in pure solvents such as water or methanol.

Here, we probe the conformational landscape of NADH accessed by changing the relative composition of a deuterated bicomponent D_2_O/DMSO-*d*_6_ solvent mixture at different temperatures by means of ^31^P NMR spectroscopy. The reason for choosing this approach is 2-fold: the pyrophosphate moiety acts as a pivot around which the folding–unfolding dynamics is expected to take place by changing the dihedral angle subtended by the P=O double bonds; thus, the ^31^P resonance and NMR relaxation rates are expected to be sensitive to the global conformation providing a cleaner biomarker for *in vivo* studies compared to ^1^H NMR spectroscopy. Second, a gradual shift of the conformational equilibrium is afforded by tuning the solvent mixture. Notably, DMSO, being an aprotic solvent, probes the influence of polarity, while D_2_O probes effects due to hydrogen bonding and hydrogen exchange.^24−27^ Our findings suggest that the two solvents induce different conformations as suggested by the opposite trends in the ps–ns tumbling rates of NADH probed by 1D spectroscopy and relaxometry studies. Changes are observed when altering the solvent mixture, and evidence is provided for suggesting the presence of an intermediate “semifolded” state.^28^

## Experimental Section

### Materials and Methods

NADH (Grade II, disodium salt, MW 709.4), DMSO-*d*_6_ (99.9 atom % D, contains 0.03% TMS, MW 84.17), D_2_O (99.9 atom % D, MW 20.03), magnesium chloride (MW 95.211), calcium chloride (MW 110.98), and aluminum chloride (MW 133.34) were acquired from Sigma-Aldrich. All samples were prepared at room temperature. Each sample comprised 30 mM NADH with or without equivalent concentration of metal chloride salts dissolved in 1000 μL of binary solvent, utilizing D_2_O and 100% DMSO-*d*_6_ mixed in varying ratios. pDs corresponding to the volume fractions of DMSO-*d*_6_ of 1 to 0 were measured as 12.2, 12.0, 11.0, 10.73, 9.84, 9.74, 8.54, 8.2, 8.14, 7.93, and 8.3. The 30 mM NADH sample in 90% DMSO-*d*_6_ and 10% D_2_O exhibited pD values of 10.45 when 30 mM MgCl_2_ was added, 11.14 for 30 mM CaCl_2_, and 9.39 for 30 mM AlCl_3_. Solutions were pipetted into a Wilmad-LabGlass 5 mm high-throughput NMR tube for NMR analysis.

### NMR Experiments

Solution NMR relaxation experiments were performed on a Bruker Avance NEO 500 MHz spectrometer with a CryoProbe Prodigy BBO probe, using Wilmad-LabGlass 5 mm high-throughput NMR tubes. *T*_1_ relaxation was measured with a standard inversion–recovery pulse sequence, and *T*_2_ relaxation was measured using a Carr–Purcell–Meiboom–Gill (CPMG) sequence. Delays varied between 2 and 5 s depending on sample conditions (temperature, pD, solvents)

#### NMR Relaxation Measurements

^31^P *T*_1_ and *T*_2_ delays were chosen such that the final two points of the *T*_1_ curves were fully recovered, and the final points for *T*_2_ curves had less than 5% of the initial intensity. *T*_1_ relaxation times were determined by employing the TopSpin 4.0.7 *T*_1_/*T*_2_ dynamics module. *T*_2_ relaxation times were determined by monoexponential fitting in MATLAB. The full width at half-maximum (FWHM) was determined by deconvolution of Lorentzian line width employing the TopSpin 4.0.7 DCON command. For each spectrum, a single scan was acquired with 10,240 size of the FID to cover a spectral window of 6579 Hz (32.5 ppm). An AU program was used to automate temperature increments and equilibration (10 min for each temperature). Autoshimming was applied continuously before and between the measurements.

#### Chemical Exchange Saturation Transfer (CEST)

**Figure 2:**
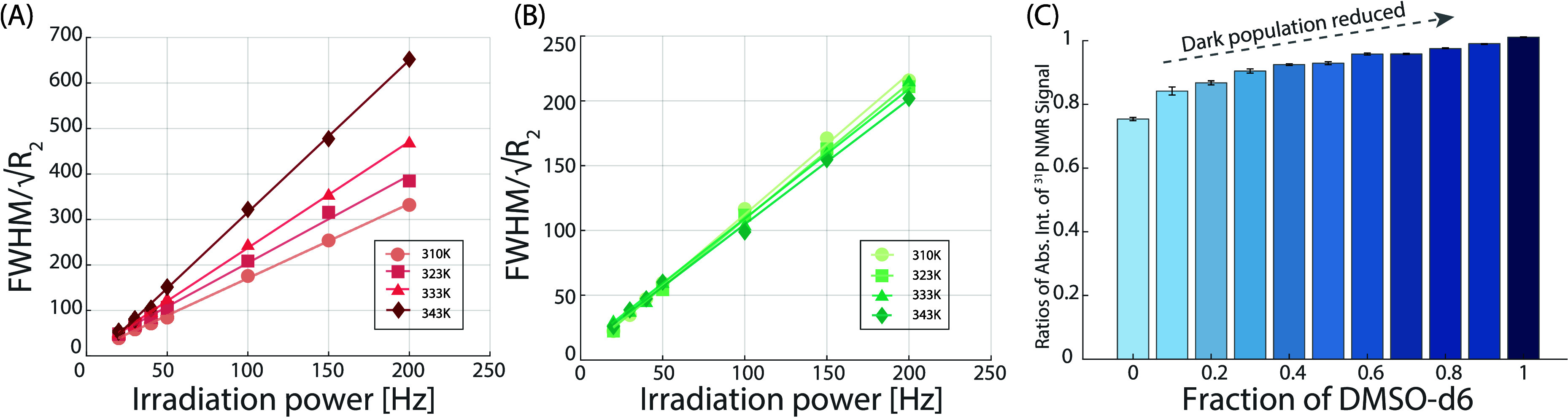
^31^P chemical exchange analysis for 30 mM NADH in pure deuterated solvents of DMSO-*d*_6_ and D_2_O. (A) Investigation of the irradiation power dependence on ^31^P CEST line widths, normalized by the square root of the spin–spin relaxation rate (FWHM/), at temperatures of 310, 323, 333, and 343 K in pure D_2_O. The increasing slope observed at these temperatures signifies an enhancement in the chemical exchange rate with increasing temperature. (B) A parallel analysis conducted in pure DMSO-*d*_6_. The uniform slopes across the studied temperatures indicate a negligible chemical exchange in this solvent. (C) Variation in the ratio of the absolute integral of the ^31^P signal from 1D NMR spectra in 30 mM NADH solutions, using binary mixed solvents of DMSO-*d*_6_ and D_2_O measured from 298 K to 343 K and adjusted for the temperature dependence as discussed in the main text.. An increasing ratio with a higher fraction of DMSO-*d*_6_ suggests that the presence of DMSO-*d*_6_ reduces the “dark” population of NADH in the solution.

^31^P CEST experiments were performed on a Bruker 500 MHz (11.7 T) NMR spectrometer equipped with a broadband observe (BBO) probe. The 90° pulse duration ranged from 11 to 12 μs depending on the ionic strength of the solution. Saturation was performed by continuous-wave (cw) irradiation of 5 s duration with field strengths of 1.16–11.6 μT (corresponding to nutation frequencies of 20–200 Hz). In Figure 2, nutation frequencies from 20 to 200 Hz for 5 s were used. The recycling delay was set to 5 s. Following cw irradiation, a 90° pulse was used for spectral readout. The temperature dependence of CEST was measured at 310, 323, 333, and 343 K with nutation frequencies of 20, 30, 40, 50, 100, 150, and 200 Hz. At each frequency offset, a single scan was acquired. The scanned offset frequency ranged from −40 to 40 ppm with various step sizes of 1, 0.25, and 0.1 ppm, as detailed in the work of Lu et al.^29,30^ As posited by Bolik-Coulon et al.,^31^ we consider the transverse relaxation rate of the spin in its ground state, denoted as *R*_2,G_. When *R*_2,G_ significantly exceeds the product of the population of excited states, *p*_E_, and the exchange rate, *k*_ex_, i.e., *R*_2,G_ ≫ *p*_E_*k*_ex_, where *p*_E_ represents the population of the excited states and *k*_ex_ is the sum of the forward and reverse exchange rates between the ground and excited states, the CEST line width can be approximated as follows: , where *T*_ex_ denotes the length of the irradiation period. Consequently, in the absence of exchange, the ratio of the chemical exchange saturation transfer line width (FWHM) to the square root of the transverse relaxation rate *R*_2_, , plotted as a function of irradiation power, should remain constant. Conversely, in the presence of exchange, this ratio is expected to increase with the exchange rate.

#### Diffusion Ordered Spectroscopy (DOSY)

^31^P DOSY measurements were acquired on a 500 MHz Bruker spectrometer with a single gradient along the *z*-axis. DOSY is an experimental that uses the pulsed field gradient NMR (PFG-NMR) technique to extract diffusion coefficients for each NMR signal present in a sample. The DOSY pulse sequence used to measure the diffusion coefficients for each sample was stegp1s. The signal attenuation as a function of the gradient strength, *g*, and the diffusion coefficient, *D*, is given as ψ(*g*,*D*) = exp(−*Dg*^2^γ^2^δ^2^(Δ – δ/3)), where γ is the gyromagnetic ratio of the nucleus, δ is the width of the gradient pulse, and Δ is the time between gradient pulses.^32^ The fitting was done automatically in Topspin 4.0.7.

All DOSY measurements were taken at 298 K. Eight scans with a 90° pulse were acquired with 10,240 data points to cover a spectral window of 6579 Hz (32.5 ppm). For diffusion measurements, the gradient strength was linearly spaced with 16 values such that the final spectrum had an intensity less than 5% of the initial one. The decays were then fit using the TopSpin 4.0.7 T1/T2 relaxation module to extract the diffusion coefficient. The delay δ ranged from 2.5 to 6 ms, and the Δ ranged from 250 to 550 ms, depending on the solution conditions. The gyromagnetic ratio, γ, of ^31^P is 10.84 × 10^7^ rad T^–1^ s^–1^. The Stokes–Einstein relation was then used to determine the hydrodynamic diameter (*d*_H_), as shown in Figure 5A,C.

#### *Ab Initio* Structures

The structures from Figure 1 (and Figure S3) were generated by geometry optimization at the DFT level of theory using ORCA software utilizing BP86 functional and Karlsruhe split-valence with polarization basis set (def2-SVP) and continuous model for water solvent. The resulting geometries converged successfully and are true local energy minima (no imaginary frequencies). In order to converge toward either “folded” or “unfolded” structure, the initial geometries were chosen to be similar to the anticipated one.

### Viscosity and Density Measurements

In this study, the kinematic viscosities (ν) of binary solvents composed of DMSO-*d*_6_ and D_2_O were measured at 25 ± 0.5 °C using an Ubbelohde viscometer type 531 01, capillary 0a (Schott-Geräte GmbH) inside a temperature-controlled room (Sterling Environments, Inc.). The binary solvent mixtures were prepared with varying ratios of DMSO-*d*_6_, ranging from 0 to 1 in volume fraction, with each sample having a total volume of approximately 20 mL. Prior to measurements, the Ubbelohde viscometer (nominal cell constant 0.005 mm^2^/s^2^) was cleaned and dried following standard procedures and then calibrated with deionized (DI) water of known viscosity at the target temperature of 25 °C to ensure precision in readings.^33^ The viscometer was filled with sample, taking special care to avoid bubble formation, and the fluid within the reservoir was equilibrated to ambient temperature for 10 min. For each measurement, a liquid volume—defined exactly between two measuring marks located directly above the capillary—flowed through the 530 μm internal diameter, 9 cm long capillary. The recorded flow time (efflux time) is directly related to the sample viscosity and was measured a minimum of three times for each sample to establish data reproducibility. The kinematic viscosity of each mixture was calculated using the standard formula ν = *Kt*, where *K* is the calibration constant of the viscometer and *t* is efflux time.

The density (ρ) of each batch of binary solvent mixtures, composed of DMSO-*d*_6_ and D_2_O of varying ratios, was determined using a digital density meter (Anton Paar DMA 4500M). Samples were injected into a U-shaped borosilicate glass tube that was vibrated at its characteristic frequency electronically. The detected eigenfrequency is related directly to the particular sample’s density. The tube was filled with DI water and air of known densities to determine the two calibration constants. All measurements were conducted at a controlled temperature of 25.0 °C, and sample volumes of 3 mL were used for each run. Density readings were recorded promptly after each sample was loaded into the instrument. To ensure accurate results, the density of each sample was measured ten times. The average of these readings was calculated to represent the density of that sample. This process was repeated for each mixture.

The dynamic viscosities (η) of the binary solvent mixtures composed of DMSO-*d*_6_ and D_2_O were calculated by utilizing the Hagen–Poiseuille equation. Using the measured density and kinematic viscosity of each mixture, the dynamic viscosity was calculated with the formula η = ρ*ν*, where the resulting unit for dynamic viscosity is Pascal-seconds (Pa·s) = 10^3^ centipoise (cP). Kinetic and end effect corrections were not considered but are assumed to be negligible due to the reasonably long efflux times. This calculation was performed for each binary solvent mixture of given sample composition. The measurements of density and calculated dynamic viscosity are shown in Figure 5B,C.

## Results and Discussion

The temperature dependence of the ^31^P NMR 1D spectra of NADH in pure solvents, D_2_O (purple-red) or DMSO-*d*_6_ (blue-green), already highlights the differences in the induced conformational preference (Figure 1A). The spectra contain a pair of strongly coupled doublets that resemble a singlet-like peak due to a small chemical shift difference between the two ^31^P nuclei. This effect is understood by inspecting the molecular structure noting the very similar electronic environment around both ^31^P atoms (see the “Unfolded” and “Folded” conformations of NADH in Figure 1). With two identical pentose rings on both sides, the first difference in molecular connectivity is reached only 7–8 bonds farther away from the pyrophosphate group. Consequently, it is expected that ^31^P spectra will preserve their singlet-like splitting patterns in both folded and unfolded states as only strong intra- or intermolecular interactions around the pyrophosphate group, such as H-bonding, can selectively alter the electronic environment (see below). The temperature dependence of the ^31^P chemical shift in the D_2_O sample (approximately −11 ppm) indicates the presence of conformational dynamics, the resulting spectra being the chemical exchange-weighted average of both U and F states. By contrast, the DMSO-*d*_6_ spectra show no temperature dependence, suggesting the presence of a single species. Previous time-resolved fluorescence studies provided convincing proof for the existence of a 50/50 folded/unfolded equilibrium of NADH in water at room temperature, while changing the solvent can lead to a preference of one state over the other. Cadena-Caicedo et al., for example, studied the folded and unfolded states of NADH by experiments in water and methanol solutions.^18^

^31^P relaxometry data (Figure 1B–D) indicate that the unfolded state is dominant in DMSO-*d*_6_ which is in line with interpretations of ^1^H spectra^34^ shown in Figure S3: as the fraction of DMSO-*d*_6_ decreases, the splitting and shifting of the H_4_^*N*^ peak suggests a dynamic equilibrium between folded and unfolded states of NADH in varying solvent environments. In the folded state, one of the H_4_^*N*^ protons is closer to the adenine ring, which alters its chemical shift due to the influence of additional electronic ring current effects. Conversely, in the unfolded state, the environment around both H_4_^*N*^ protons is more similar, leading to less pronounced chemical shift differences, which was observed in pure DMSO-*d*_6_. The longitudinal (*R*_1_) and transverse (*R*_2_) relaxation rates of ^31^P in the DMSO-*d*_6_ sample follow the expected temperature dependence behavior: *R*_1_ describes a concave parabola with a maximum corresponding to the condition τ_*C*_ω_0_ = 1, where τ_*C*_ is the rotational correlation time of the molecule and ω_0_ is the Larmor frequency of the ^31^P nucleus (201.86 MHz for a 11.7 T magnet); *R*_2_ decreases monotonically as τ_*C*_ decreases (temperature increases). For the D_2_O sample, the *R*_1_ values decrease with increasing temperature, suggesting that the maximum value can be reached by further cooling the sample. The fact that the two solvents impose different temperature values for the τ_*C*_ω_0_ = 1 condition can be accounted for by the larger viscosity of DMSO-*d*_6_ and the different hydrodynamic radii of the folded and unfolded states (see below). Most surprisingly, the transverse rate increases with increasing temperature for the water-abundant mixtures, a phenomenon which further points toward chemical exchange between different states, as also described for other organic phosphates in very recent studies.^29,30^

Another technique that is often use to probe the existence of low-concentration states (“dark states”) in exchange with a more abundant species is chemical exchange saturation transfer (CEST).^35,36^ Here, we employed the method from ref (37) and obtain further evidence to support our finding regarding NADH dynamics in D_2_O and DMSO-*d*_6_ (Figure 2A,B). The dispersion profile of ^31^P CEST line widths as a function of irradiation power at different temperatures (normalized by the square root of the spin–spin relaxation rate (FWHM/) indicates the presence of chemical exchange for the water sample, but not for the DMSO-*d*_6_ sample.

**Figure 3:**
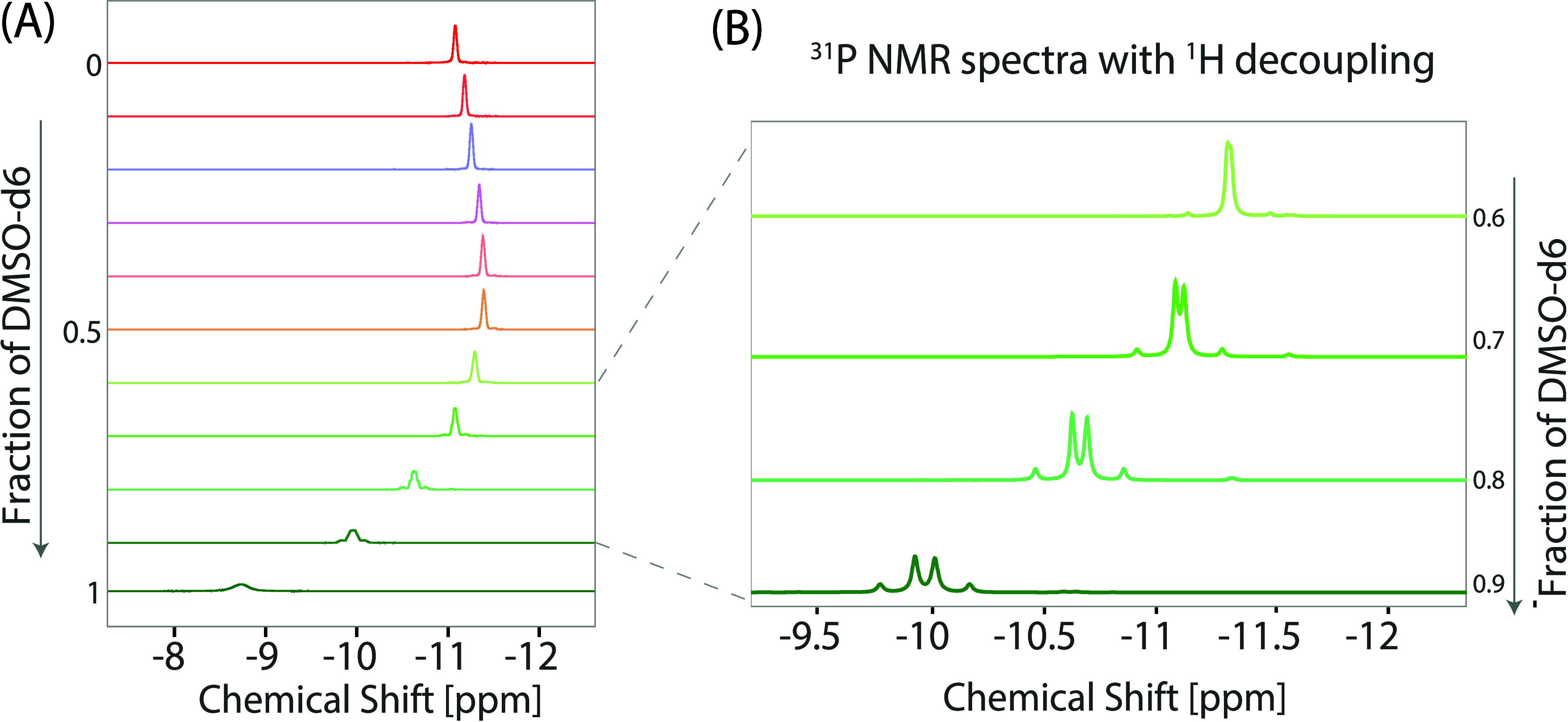
^31^P NMR results of 30 mM NADH in binary mixed solvents of DMSO-*d*_6_ and D_2_O at 298 K. (A) 1D NMR spectra of 30 mM NADH in binary mixtures, with varying DMSO-*d*_6_ fractions ranging from 1 (pure DMSO-*d*_6_) to 0 (pure D_2_O), displayed in descending order (bottom to top) with increments of 0.1. These spectra exhibit line narrowing as the fraction of DMSO-*d*_6_ decreases. Significantly, ^31^P peak splitting is observed at specific concentrations, particularly at 90%, 80%, and 70% DMSO-*d*_6_. The ^31^P chemical shift was calibrated with 85% H_3_PO_4_. (B) ^31^P NMR spectra with ^1^H decoupling, highlighting the evolution of peak patterns: a doublet of doublets is observed at DMSO-*d*_6_ concentrations of 90%, 80%, and 70%, which then disappears at a concentration of 60% DMSO-*d*_6_.

As a next step, we investigated the impact of the bicomponent mixture composition in order to further probe the conformational landscape of NADH: starting from pure DMSO-*d*_6_ solvent, where only the U state is assumed to be prevalent, gradually increasing the D_2_O ratio impacts several observables such as the ^31^P chemical shift, the splitting pattern, the relaxation rates, and the diffusion constant. These changes are interpreted in terms of the water’s impact on NADH conformation which promotes chemical exchange between U and F states. Notably, a nonmonotonic upfield shift of ^31^P resonances is observed (Figure 3A) with extreme values at −8.7 ppm (in pure DMSO-*d*_6_) and −11 ppm (in pure D_2_O). In parallel, for a specific range of solvent ratios (Figure 3B), the resulting splitting patterns point toward an enhanced difference in terms of electronic environments for the two ^31^P nuclei leading to a larger chemical shift difference. This effect can be attributed to intramolecular interactions in the form of H-bonds involving pyrophosphate’s oxygen atoms and nicotinamide protons or intermolecular interactions with D_2_O–DMSO-*d*_6_ complexes. Previous MD studies^7,28^ point toward the former case while stable water–DMSO complexes have also been reported in the literature.^24,26,27^ Noting that ^31^P spectra show a temperature and mixture composition dependence and considering the hypothesized presence of invisible “dark states”, we measured the ratio of peak integrals from 298 to 343 K for different D_2_O/DMSO-*d*_6_ fractions (Figure 2C) and adjusted for the temperature dependence of the magnetization (1/*T* Curie factor). In the case of pure DMSO-*d*_6_ solvent, there is no variation of the resulting peak integral, suggesting that all ^31^P spins in the sample correspond to the same chemical species, i.e., the large peak corresponding to the unfolded state without exchange with a hidden pool. Adding water leads to a drop in the peak integral from low to high temperatures, proving that the ^31^P spins are distributed among multiple species, i.e., the abundant unfolded and “dark” folded states giving rise to chemical exchange-averaged spectra.

**Figure 4:**
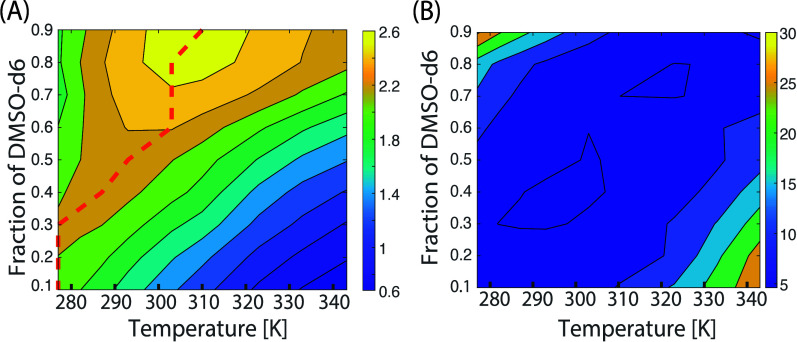
^31^P relaxation characteristics of 30 mM NADH in binary mixed solvents of DMSO-*d*_6_ and D_2_O, with DMSO-*d*_6_ fractions ranging from 0.1 to 0.9, across temperatures from 277 to 343 K. (A) Spin–lattice relaxation rate (*R*_1_) as a function of temperature. The dashed line indicates the maximum *R*_1_ values observed at each solvent fraction. (B) Spin–spin relaxation rate (*R*_2_) as a function of temperature, showing an increase in *R*_2_ correlating with both higher fractions of DMSO-*d*_6_ and increased temperature.

**Figure 5:**
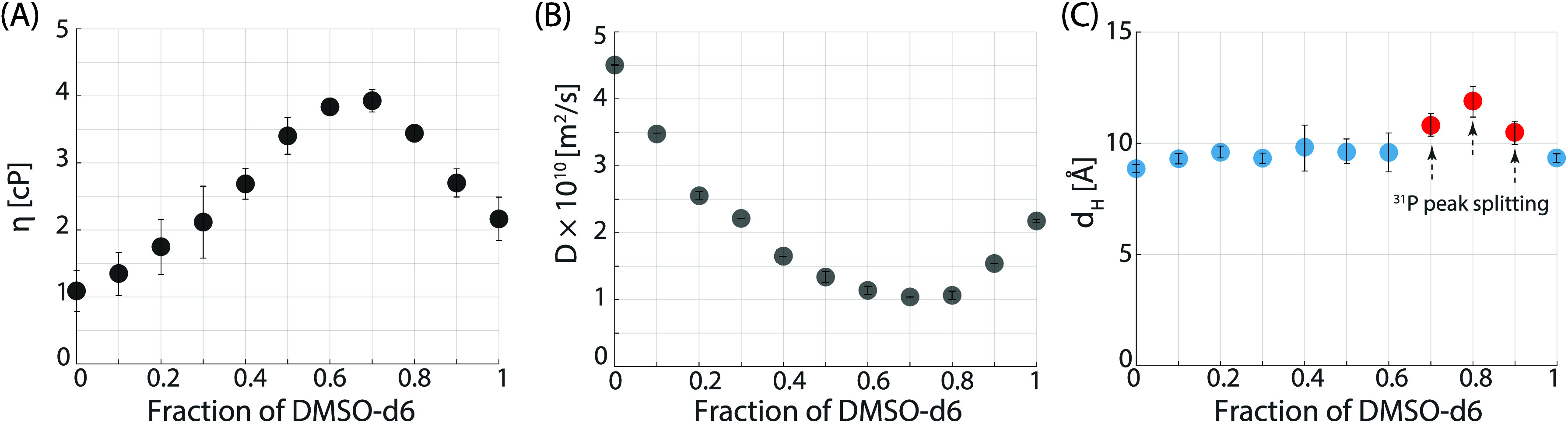
Comprehensive analysis of dynamic viscosity (η), diffusion coefficient (*D*), and hydrodynamic diameter (*d*_H_). (A) Dynamic viscosity measurements of binary mixed solvents at 298 K. (B) ^31^P DOSY results for 30 mM NADH in the binary mixed solvents. (C) Hydrodynamic diameter extracted from the diffusion coefficients in (B) for 30 mM NADH in the binary mixed solvents, providing a quantitative measure of the molecular size of NADH in various solvent compositions. Arrows indicate specific points where ^31^P peak splitting from a singlet to a doublet of doublets is observed, specifically at 70%, 80%, and 90% DMSO-*d*_6_ concentrations.

The relaxation data shown in Figure 4 highlight two different phenomena: first, due to the nonlinearity of viscosity as a function of D_2_O/DMSO-*d*_6_ composition (see Figure 5A), the rotational correlation time, and thus relaxation rates, do not always follow a monotonic trend when changing the solvent fraction. The maximum value of ^31^P *R*_1_ shifts to higher temperatures (red dashed line) due to the slowdown of molecular tumbling as the fraction of the more viscous solvent increases. Based on previous measurements from literature, we can predict the viscosity of each mixture composition over the temperature range used in our experiments (Figure S1). Starting from these values, we use a simple relaxation model based on the common spin interactions that can impact ^31^P magnetization in order to fit the measured *R*_1_ and *R*_2_ data (see Part 1 of the Supporting Information). For the case of pure DMSO-*d*_6_ at room temperature, a rotational correlation time of τ_*C*_ = 1/ω_0_ ≈ 0.8 ns is estimated, which corresponds to a hydrodynamic radius of 7.24 Å (using Stokes’ law) for the unfolded state. The transverse relaxation rate *R*_2_ follows a monotonic decrease with the DMSO-*d*_6_ fraction and increasing temperature (for the case of pure DMSO-*d*_6_) while mixtures with abundant water content show a clear deviation from the expected behavior. Normally, as temperature and tumbling rate increase, *R*_2_ values should decrease, as predicted by our model. Our results can be explained in terms of an increasing chemical exchange rate between the two (or more) U/F chemical sites which have been proposed in previous reports.^9,17^ Thus, the two maxima shown in Figure 4B can be explained by a decrease of the tumbling rate (due to low temperature and DMSO’s higher viscosity) and an increased chemical exchange rate for the water-rich mixtures at high temperatures.

To gain insight into the size of the U/F states, we conducted ^31^P DOSY experiments as well as independent viscosity measurements (Figure 5). Using the Stokes–Debye–Einstein equation, we were able to infer the hydrodynamic diameter for NADH at room temperature for all D_2_O/DMSO-*d*_6_ mixtures (Figure 5C). The values are constant, around 10 Å, with a clear increase for certain compositions (red dots) which correlate with the different splitting pattern observed in the ^31^P 1D spectra (Figure 3B). These findings support the existence of an intermediate “semifolded” state where the two ^31^P nuclei have different chemical environments due to either intramolecular H bonding or solvent interactions, while the molecule undergoes a significant change of the overall conformation.

**Figure 6:**
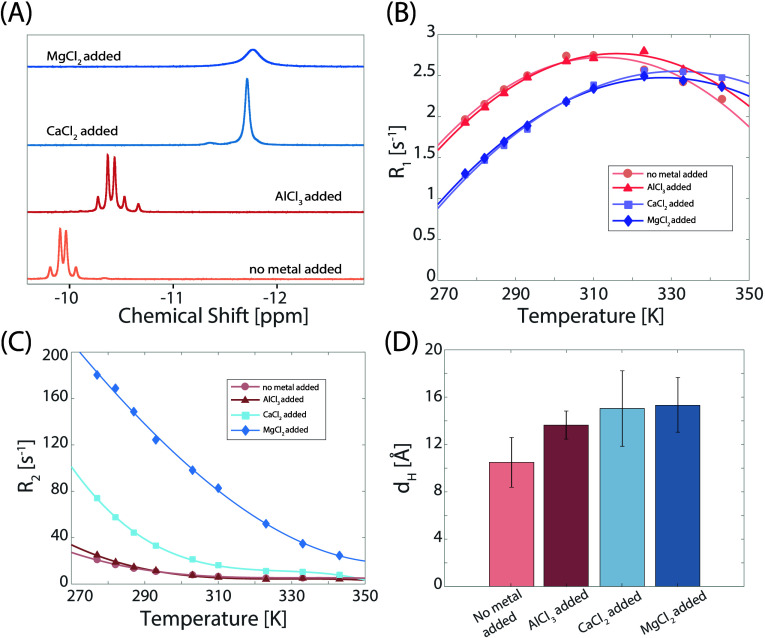
^31^P NMR analysis of 30 mM NADH in 90% DMSO-*d*_6_ and 10% D_2_O, with and without 30 mM metal chloride salts. (A) ^31^P 1D NMR spectra variations: without metal chloride salts, the NADH peak appears as a doublet of doublets. Addition of 30 mM AlCl_3_ results in slight shielding (0.46 ppm) but retains the doublet of doublet structure. With 30 mM CaCl_2_, the peak transitions to a singlet with greater shielding (approximately 1.77 ppm), similar to the case with 30 mM MgCl_2_, though the MgCl_2_ added one has a broader line width. (B) Spin–lattice relaxation (*R*_1_) behavior: NADH shows a local maximum in *R*_1_ both with and without metal chloride salts added. The *R*_1_ profiles of NADH without metal and with AlCl_3_ track each other. The addition of CaCl_2_ or MgCl_2_ alters the *R*_1_ profile, delaying the appearance of the local maximum. (C) Spin–spin relaxation (*R*_2_) behavior: all *R*_2_ curves exhibit a monotonic decrease. The profiles in the absence of metal chloride salts and with AlCl_3_ are similar, while the addition of MgCl_2_ or CaCl_2_ significantly enlarges *R*_2_. (D) Hydrodynamic diameter (*d*_H_) extracted from the measured diffusion coefficients and dynamic viscosity in Figure 5A; all metal chloride salts increase NADH size, in the order of AlCl_3_ < CaCl_2_ < MgCl_2_.

Further investigation of this conformation was conducted for a D_2_O/DMSO-*d*_6_ = 1/9 sample by adding metal ions in an attempt to induce either one of the known folded or unfolded states upon chelation. Figure 6A shows the different ^31^P 1D spectra. Little effect is observed for Al^3+^ addition, when compared to addition of Ca^2+^ or Mg^2+^. The upfield shift induced by addition of the latter two indicates a preference for the folded state (similar to increasing the water content) which is paralleled by the collapse of the pair of doublets into a singlet-like pattern. The temperature profile of the longitudinal relaxation rate further suggests a large effect of Ca^2+^ and Mg^2+^ binding by shifting the maximum *R*_1_ value to higher temperature which translates to an increase of the molecular size. This effect can be accounted for by the coordination of extra solvent molecules to the metal which slows down the overall molecular tumbling as also reflected by the increase in *R*_2_ and hydrodynamic radii for the Ca^2+^/Mg^2+^ complexes (Figure 6C,D).

Based on our findings about NADH’s different conformations under different solvent conditions in this study, we expect that such phenomena should be taken into consideration in the biological context. DMSO has been widely used in chemistry, biology, and medicine by serving as an efficient cell cryoprotectant as well as a carrier for drug therapy and for the *in vivo* administration of water-insoluble compounds.^38,39^ Our observations suggest that NADH may adopt various conformations depending on the environment and the presence of cations, which could significantly impact its enzymatic binding. Enzymes that interact with NADH could have different affinities and activities depending on whether NADH is in a fully folded, semifolded, or extended conformation. This conformational flexibility might be critical in biological systems where NADH participates in numerous metabolic pathways. Additionally, metal ion concentration changes in cellular environments often play roles of stabilizing certain biomolecular structures or facilitating enzymatic reactions. The presence of metal cations might stabilize specific NADH conformations, thereby modulating its biological activity. Understanding these conformational dynamics in different environments enhances our knowledge of NADH’s functionality and its role in cellular metabolism.

## Conclusion

As described in previous literature, both NAD^+^ and NADH can exist in a chemical equilibrium between two conformations, folded and unfolded, which in turn dictates the rate of binding to enzymes where typically the latter state is preferred. By means of ^31^P NMR, we have explored the conformational landscape of NADH in a bicomponent mixture of deuterated water and dimethyl sulfoxide showing strong solvent-induced changes of the 3D structure. Several observable characteristics for ^31^P nuclei, such as chemical shifts and relaxation rates, can be used to assess the conformational state of NADH. Potential applications may even include *in vivo* studies where ^31^P NMR could be a better alternative than ^1^H NMR due to the reduced spectral overlap. Relaxometry and diffusion studies offer a glimpse into the overall tumbling rate and size of NADH conformers pointing toward a multisite chemical exchange equilibrium involving folded, semifolded, and unfolded states.

## Supporting Information Available

Viscosity profiles for DMSO–D_2_O mixtures across different temperatures and compositions; comparison of theoretical and actual *R*_2_/*R*_1_ relaxation ratios under varying conditions, indicating discrepancies; ^1^H NMR spectral changes in NADH due to different DMSO–D_2_O solvent compositions, reflecting conformational shifts (PDF)
