# Supplementary material for: Conformational Landscape of NADH and Ion Binding in Water/DMSO Mixtures via 31P NMR Spectroscopy
Source: J Phys Chem B. 2024 Jul 17;128(35):8504–11. doi: 10.1021/acs.jpcb.4c03162 (PMC11382270; doi:10.1021/acs.jpcb.4c03162)
Supplement: Supplementary file 1 — jp4c03162_si_001.pdf [file jp4c03162_si_001.pdf]

Supporting Information: Conformational Landscape of NADH and Ion-binding in Water/DMSO Mixtures via ^{31}P NMR Spectroscopy

Jiaqi Lu,[†] Florin Teleanu,[†,‡,¶] Huijing Zou,[†] Chengtong Zhang,[†] Andrew Hollingsworth,[§] and Alexej Jerschow[*,†]

†*Department of Chemistry, New York University, New York, NY 10003, United States*

‡*Interdisciplinary School of Doctoral Studies, University of Bucharest, Bucharest 010041, Romania*

¶*Biophysics and Biomedical Application Laboratory, Extreme Light Infrastructure Nuclear Physics, IFIN-HH, Măgurele 77125, Romania*

§*Department of Physics, New York University, New York, NY 10003, United States*

E-mail: alexej.jerschow@nyu.edu

Fitting ^{31}P relaxation rates over the solvent fraction and temperature range

In order to better understand the NADH dynamics in solution, we attempted to fit the relaxation rates over both the temperature and solvent mixture dimensions. From simulations it is found that dipolar interactions do not contribute significantly to the relaxation rates of ^{31}P magnetization. Based on the predicted molecular structure of NADH, the ^{31}P-^{31}P and ^{1}H-^{31}P dipole-dipole interactions (arising from the neighboring methylene groups) lead to small dipolar coupling constants of around 7 kHz and 13 kHz, respectively. These interactions contribute with only 0.02-0.1 s^{-1} and 0.15-0.5 s^{-1} to the longitudinal and transverse relaxation rates of ^{31}P magnetization on a rotational dynamic range of 0.1-5 ns (see attached Spinach[S1] notebook). The dominant relaxation mechanism for the ^{31}P spin pair is given by the anisotropy of the chemical shielding tensor at individual nuclei. The expressions for the two relaxation rates are:[S2]

$$R_1 = (\gamma \Delta\sigma B_0)^2 J(\omega_0) \tag{1}$$

$$R_2 = \frac{1}{6}(\gamma \Delta\sigma B_0)^2 (4J(0) + 3J(\omega_0)), \tag{2}$$

where γ is the gyromagnetic ratio of ^{31}P (10.84×10^7 rad T^{-1} s^{-1}), $\Delta\sigma$ is the chemical shielding anisotropy (CSA), B_0 is the main magnetic field and $J(\omega) = \frac{2}{5}\frac{\tau_C}{1+\tau_C^2\omega^2}$ is the spectral density function. Our experiments run at different temperature and solvent mixture composition imply a significant change of viscosity (η) which impacts the rotational correlation time (τ_C) through the Stokes-Debye equation: $\tau_C = \frac{4\pi\eta r_H^3}{3kT}$. Thus, we made use of previous viscosity measurements from literature[S3] and ran a bidimensional interpolation in temperature and DMSO volume fraction (See Figure S1).

To estimate the hydrodynamic radius from relaxation data, we derived the rotational correlation time using $\tau_C = \frac{1}{\omega_0}$ at the maximum value of the longitudinal relaxation rate R_1, for the 0.9/0.1 DMSO/D_2O sample at 310K (Figure 4). In this way, we obtained $\tau_C = 0.8$ ns

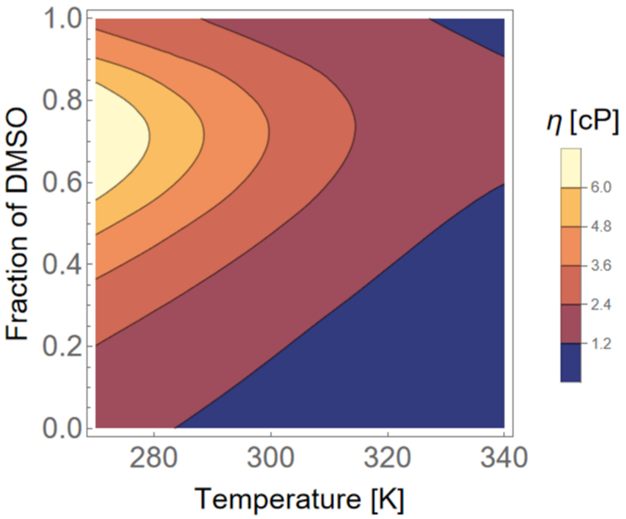

Figure S1: Contour plot of interpolated viscosity profiles for binary DMSO-D_2O system at different temperatures and volume fractions (raw data from Ref S3).

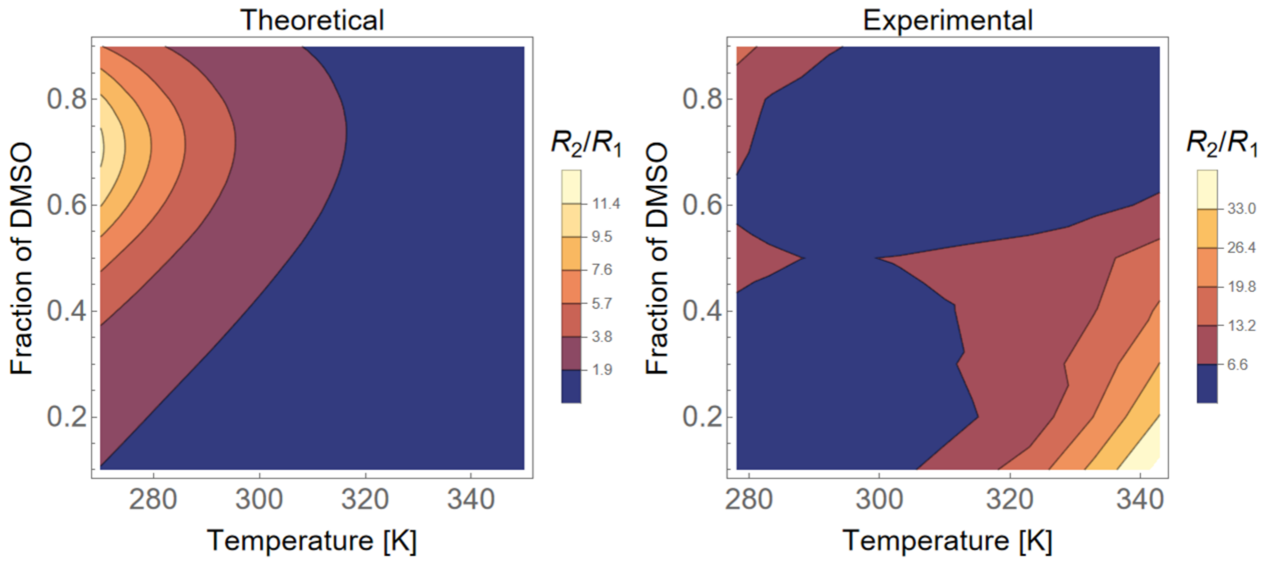

Figure S2: Contours plot of theoretical and experimental values of R_2/R_1 at different temperatures and volume fractions of DMSO.

which corresponds to a hydrodynamic radius of 7.24 Å using the Stokes-Debye equation with an interpolated viscosity $\eta = 4.8cP$ (Figure S1). Knowing the viscosity both as a function of temperature and solvent mixture η(T, Fraction of DMSO) and initially assuming that the hydrodynamic radius is constant throughout, the spectral density $J(\omega)$ is completely determined across our experimental conditions. From Eqs (1) and (2), we notice that the quantity R_2/R_1 is independent of the unknown CSA interaction strength which renders it a more robust evaluation of the tumbling rate. In Figure S2, we compare the predicted and experimental values of the R_2/R_1 ratio and observe significant deviations. These discrepancies can be interpreted in terms of fluctuations of the hydrodynamic radii and/or the presence of chemical exchange. Both assumptions are in agreement with the conformational dynamics hypothesis described in the main text, as increases in water content and temperature alter the conformational equilibrium.

^{1}H NMR signature of conformation-sensitive nicotine protons

Figure S3 presents the ^{1}H NMR spectra of 30 mM NADH in binary mixed solvents of DMSO-d_6 and D_2O at a temperature of 310 K. The purpose of this experiment is to investigate the effects of solvent composition on the NMR chemical shifts and multiplicity of the H_4^N signal, and demonstrate the composition of NADH conformations in varying solvent environments.

The spectra show results from a series of experiments with varying volume fractions of DMSO-d_6, ranging from 1 (100% DMSO-d_6) to 0 (100% D_2O). These fractions change incrementally by 0.1, and the spectra are ordered from top (pure DMSO-d_6) to bottom (pure D_2O) in the figure. A significant change in the chemical shift is observed where the H_4^N peak moves from 3.79 ppm in pure DMSO-d_6 to 3.43 ppm in pure D_2O. Additionally, the peak splitting in a sample with a higher proportion of D_2O, where the H_4^N peak appears as a multiplet rather than a singlet, suggests a change in the structural conformation of NADH.

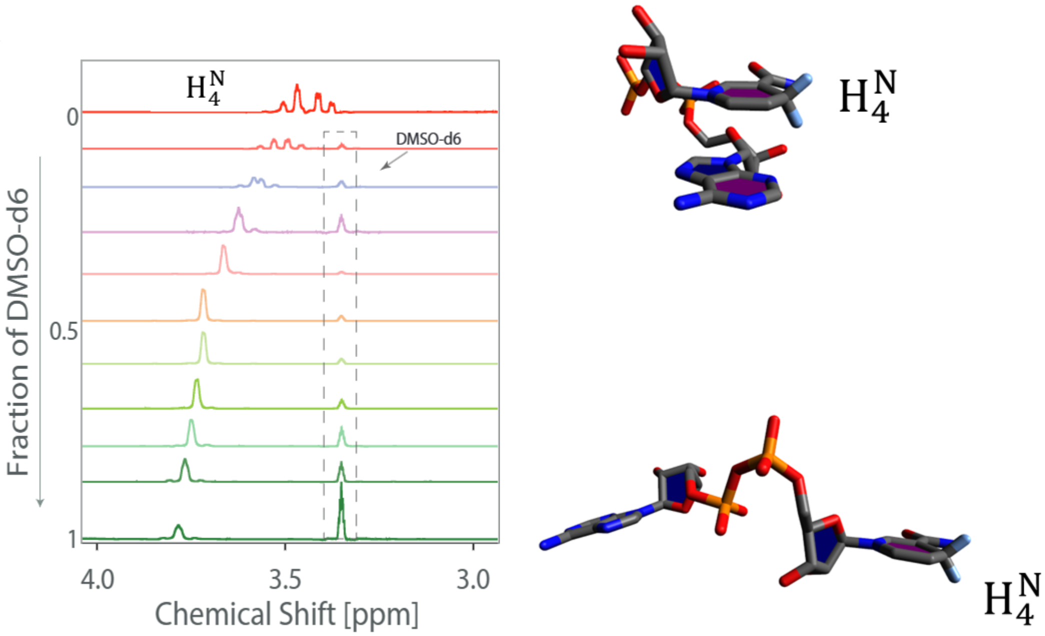

Figure S3: ^{1}H NMR spectra of 30 mM NADH in binary mixed solvents of DMSO-d_6 and D_2O at 310 K. The figure displays 1D NMR spectra of 30 mM NADH in varying binary mixtures, with DMSO-d_6 volume fractions ranging from 1 (pure DMSO-d_6) to 0 (pure D_2O), shown in ascending order from top to bottom, with incremental changes of 0.1. The $H_4{}^N$ peak is observed to split into a multiplet and shift from downfield 3.79 ppm to upfield 3.43 ppm with a decreasing volume fraction of DMSO-d_6. The structures of the folded and unfolded states highlights the reason for the enhanced chemical shift different between the two $H_4{}^N$ protons (pale blue): in the folded state, one proton is closer to the adenine ring and experience the additional electronic current, while in the unfolded state, both protons have very similar environments. Peaks within the dashed rectangular box represent the DMSO-d_6 peak, while the left-sided peaks correspond to the $H_4{}^N$ signals. The ^{1}H peak of $H_4{}^N$ shows as a singlet peak when NADH is unfolded, while it split into a pair of multiplet when there is folded conformation exists.

As the environment becomes less polar (higher DMSO-d_6 content), NADH tends to adopt an unfolded conformation where the magnetic environment of the H_4^N protons becomes more equivalent.

The splitting and shifting of the H_4^N peak suggests a dynamic equilibrium between folded and unfolded states of NADH in varying solvent environments. In the folded state, one of the H_4^N protons is closer to the adenine ring, which alters its chemical shift due to the influence of additional electronic currents. Conversely, in the unfolded state, the environment around both H_4^N protons is more similar, leading to less pronounced chemical shifts. The dashed rectangular boxes in the figure denote peaks corresponding to DMSO-d_6, serving as a reference for solvent presence. The left-sided peaks in each spectrum relate directly to the H_4^N signals, highlighting the changes across the series.

This set of spectra provides significant insights into the solvation effects and conformational dynamics of NADH, which are critical for understanding its behavior in biological systems and its interactions in different solvent environments.

References

(S1) Hogben, H. J.; Krzystyniak, M.; Charnock, G. T. P.; Hore, P. J.; Kuprov, I. Spinach – A software library for simulation of spin dynamics in large spin systems. *J. Magn. Reson.* **2011**, *208*, 179–194.

(S2) Kowalewski, J.; Maler, L. *Nuclear Spin Relaxation in Liquids: Theory, Experiments, and Applications, Second Edition*; CRC Press LLC, 2019.

(S3) Cowie, J. M. G.; Toporowski, P. M. Association in the binary liquid system dimethyl sulphoxide – water. *Can. J. Chem.* **1961**, *39*, 2240–2243.
